# *WTH3* is a direct target of the p53 protein

**DOI:** 10.1038/sj.bjc.6603724

**Published:** 2007-04-10

**Authors:** K Tian, Y Wang, H Xu

**Affiliations:** 1 Department of Biochemistry and Cell Biology, State University of New York at Stony Brook, Stony Brook, NY, USA; 2 Laboratory of Pathobiology, Jilin University, Changchun, The People's Republic of China

**Keywords:** WTH3 gene, p53-response element, multidrug resistance, apoptosis

## Abstract

Previous results showed that overexpression of the *WTH3* gene in multidrug resistance (MDR) cells reduced *MDR1* gene expression and converted their resistance to sensitivity to various anticancer drugs. The *WTH3* gene promoter was found to be differentially regulated in paired MDR *vs* non-MDR MCF7 cells owing to epigenetic modifications and transcription factor modulations. To understand further the mechanisms that govern *WTH3*'s differential expression, we uncovered a p53-binding site in its promoter, which indicated that *WTH3* could be regulated by the *p53* gene. This hypothesis was then tested by different strategies. The resulting data revealed that (1) the *WTH3* promoter was upregulated by the *p53* transgene in diverse host cells; (2) there was a correlation between *WTH3* expression levels and *p53* gene status in a cell line panel; (3) a *WTH3* promoter region was directly targeted by the p53 protein *in vitro* and *in vivo*. In addition, overexpression of the *WTH3* gene promoted the apoptotic phenotype in host cells. On the basis of these findings, we believe that the negative role played by the *WTH3* gene in MDR development is through its proapoptotic potential that is regulated by multiple mechanisms at the transcription level, and one of these mechanisms is linked to the *p53* gene.

Multidrug resistance (MDR) is a fatal event encountered during cancer chemotherapy (Chen *et al*, 1986; Gros *et al*, 1986a, 1986b; Cole *et al*, 1992; Robinson *et al*, 1997; Smyth *et al*, 1998; Johnstone *et al*, 1999). To understand better MDR development, we employed the methylation sensitive-representational difference analysis technique (Yuan *et al*, 1999; Shan *et al*, 2000, 2002a) to study DNA hypermethylation events in a human MDR breast cancer cell line, MCF7/AdrR, as compared to its parental line, MCF7/WT. As a result, the *WTH3* gene was discovered. The gene product is homologous to the *Rab6* and *Rab6c* genes that belong to the *ras* super family and encode small G proteins (Zahraoui *et al*, 1989; Goud *et al*, 1990; Echard *et al*, 1998, 2000; Shan *et al*, 2000, 2002a). Similar to the *Rab6*s, *WTH3* is a housekeeping gene and its product is capable of binding to GTP molecules (Tian *et al*, 2005b). However, unlike the Rab6s that reside in the Golgi network, most of WTH3 locates in the cytoplasm and to a less degree in the nuclei. This disparity could be due to WTH3's lack of a cysteine at its C terminus for geranyl–geranylation, a necessary post-translational modification for membrane attachment (Barbacid, 1987). Previous studies found that the *WTH3* gene was downregulated in MDR cell lines, MCF7/AdrR and MES-SA/Dx5 (a human uterine sarcoma cell line), and by introducing it back into those lines caused downregulation of *MDR1* gene expression and reversed their MDR phenotypes to various anticancer drugs (Shan *et al*, 2002a; Tian *et al*, 2005b). In addition, our research also revealed that hypermethylation (an epigenetic modification event in mammals) of the *WTH3* promoter and transcription factor modulation were involved in its differential expression in MCF7/AdrR *vs* MCF7/WT cells (Tian *et al*, 2005b). Furthermore, the hypermethylation event was also observed in primary drug-resistant breast cancer cells (Tian *et al*, 2005a). Taken together, our data supported the notion that *WTH*3 could play an important role in MDR development.

To understand further the mechanisms involved in *WTH3*'s differential expression in MDR cells, the Patch Search Program was utilised to look for consensus sequences in the gene promoter for existing transcription factors. As a result, several candidate motifs were implicated, one of which was a p53-binding domain, although it contained five mismatched base pairs (bp) as compared to the typical p53-binding consensus sequence (5′-RRRCWWGYYY(N=0–13)RRRCWWGYYY-3′) (Lowe, 1995). Consequently, we named it p53M. The *p53* gene product is a transcription factor that functions as a tumour suppressor and plays a pivotal role in apoptosis and cell cycle arrest (Lowe, 1995; Aas *et al*, 1996; Righetti *et al*, 1996). Various mutations of *p53* are associated with human cancers and the onset of MDR in a broad field of solid and haematological malignancies (Ogretmen and Safa, 1997; Schmitt and Lowe, 1999; Smith *et al*, 2003; Norbury and Zhivotovsky, 2004; Pommier *et al*, 2004; Kim, 2005; Steele and Lane, 2005). Identification of a potential p53-binding motif in the *WTH3* promoter suggested that its activity could be regulated by *p53*. To test this hypothesis, *WTH3* gene promoter function under the influence of the *p53* transgene was evaluated by luciferase assays. In addition, the correlation between *WTH3* expression levels in 11 cell lines with defined *p53* status was examined. Owing to the mismatches in p53M, instead of using the targeted-deletion strategy, we generated serial deletion mutants to determine the p53-response region in the *WTH3* promoter. Next, the physical interaction between the defined response region and the p53 protein was examined by the electrophoretic mobility shift assay (EMSA) and chromatin immunoprecipitation (ChIP) approach. The resulting data suggested that the *WTH3* gene was a direct target of the p53 protein. This information led us to evaluate the possible participation of *WTH3* in promoting apoptosis.

## MATERIALS AND METHODS

### Cell lines and doxorubicin (DOX) treatment

MCF7/AdrR (p53 del 126–132), MCF7/WT (WT p53) (Ramljak *et al*, 2005), MES-SA/Dx5 and its parental cell line, MES-SA (ATCC.), were grown under the conditions as described (Shan *et al*, 2002a). Hs578T (p53 V157F), MDA-MB-231 (p53 R280K), MDA-MB-435 (p53 G266E), MDA-MB-436 (mutated p53), MDA-MB-468 (p53 R273H), T47D (p53 L194F) and SKBr3 (p53 R175H) (Camps *et al*, 1990; Elstner *et al*, 1995; Nieves-Neira and Pommier, 1999) (gifts from Dr Moll M Ute, Department of Pathology; and Dr Cao Jian Division of Hematology, SUNYSB), were grown at 37°C with 5% CO_2_ in Dulbecco's modified Eagle's medium medium with 10% foetal calf serum (FCS), 100 *μ*g/ml streptomycin and 100 U/ml penicillin. HEK293 (Human primary embryonic kidney cells, ATCC), and Hela cells were grown at 37°C with 5% CO_2_ in Roswell Park Memorial Institute 1640 culture medium with 10% FCS, 100 *μ*g/ml streptomycin and 100 U/ml penicillin. To induce *p53* expression, MCF7/WT cells were treated with 1 *μ*M of DOX for 20 h.

### Construction of recombinant DNA

Detailed information about generating the pcDNA3.1/WTH3 and pGL/WTH3P constructs containing the coding region and *WTH3* promoter, respectively, were described previously (Shan *et al*, 2002a; Tian *et al*, 2005b). Six deletion mutants of the *WTH3* promoter were created by polymerase chain reaction (PCR) amplification using pGL/WTH3P as the template. Sense primers for deletion 1 (−540 to −1), 2 (−453 to −1), 3 (−396 to −1), 4 (−289 to −1), 5 (−194 to −1) and 6 (−116 to −1) were 5′-AGAG GTACCCACCGCACCATTGTTTTTAGTAC-3′, 5′-AGAGGTACCCGCACTCAGCAGGTTGGGC-3, 5′-AGAGGTACCTGAGAGATCCCGGATACATCTGC-3′, 5′-AGAGGTACCCAAAGCACACCCCTGGCTC-3, 5′-AGAGGTACCGGCGG CTGCCAGTCTGTG-3′ and 5′-AGAGGTACCGGGGCGCAGAGAGCTCGG-3, respectively. The anti-sense primer for all the mutants was 5′-GAAGATCTTCGTGGAACTAGAGGAGCTGTCGCC-3′. Each primer pair contained *Kpn*I and *Bam*HI restriction enzyme sites for cloning the PCR fragment into the pGL3 vector to create pGL/WTH3P-d1, -d2, -d3, -d4, -d5 and -d6. The correct sequence of each construct was verified by sequencing (Genewiz Inc., South Plainfield, NJ, USA). Wild-type *p53* in pcDNA/P53 and the mutated *p53* gene in pcDNA/P53R249S, which did not contain *trans*-element activity, were gifts from Dr Moll M Ute.

### Transient transfection and luciferase assays

To determine whether the *WTH3* promoter was regulated by *p53*, pGL/WTH3P was cotransfected with pcDNA/P53, pcDNA/P53R249S (negative control) or pcDNA/3.1 (negative control) into MCF7/WT and HEK293 cells. In brief, 0.2 *μ*g of each construct were transfected along with 0.1 *μ*g of pCMV/*β*-galactosidase when the cells (seeded onto 24-well plates) reached 50–70% confluence. After 24 h, luciferase and *β*-galactosidase activity was measured using the Luciferase Assay System and Beta-Glo™ Assay System (Promega, Madison, WI, USA) according to the manufacturer's instruction. Luciferase activities of transfectants were compared after normalising their *β*-galactosidase activities and protein concentrations. To determine *p53*s influence on endogenous *WTH3* gene expression, pcDNA/P53 or the empty vector was transiently transfected into Hela and MCF7/AdrR cells. After 24 h, RNAs were isolated from the cells for semi-quantitative reverse transcriptase (SQRT)-PCR analyses.

### SQRT-PCR and Western blot

Total RNAs were isolated from cell lines, transfectants and the corresponding negative controls by the High Pure RNA Isolation Kit (Roche, Indianapolis, IN, USA). Semi-quantitative reverse transcriptase-polymerase chain reaction was performed using the Titan One Tube RT-PCR system based on the manufacturer's protocol (Roche). The sense and anti-sense primers for *WTH3* and *GAPDH* were described previously (Shan *et al*, 2002a). The PCR and quantification of PCR products were performed as described (Shan *et al*, 2000, 2002a; Tian *et al*, 2005a, 2005b). To evaluate WTH3 protein levels in the cell lines, the protein concentrations of cell lysates were determined by absorbance measured at 280 nm and the bicinchoninic acid protein assay reagent kit (BCA Kit, Pierce, Rockford, IL, USA). A total of 100 *μ*g of each cell lysate was loaded onto triplicate 12% sodium dodecyl sulphate polyacrylamide gel electrophoresis (SDS-PAGE) gels for Western analyses as described previously (Shan *et al*, 2002b) using the antibodies for WTH3 (1 : 200 dilution) (Research Genetics, Huntsville, AL, USA), Rab6 (1 : 2000 dilution) and MDR1 (1 : 30) (Santa Cruz, Santa Cruz, CA, USA).

### EMSA and super-EMSA

Electrophoretic mobility shift assay were performed using the purified p53 protein (Santa Cruz) or and probe (P49) that covered the region from −282 to −330 in the *WTH3* promoter (gagccgggtgcggaaggagggaacg[gCCctagcct/TggGaagccA]aagc-3′) and contained the putative p53-response element, p53M (bracketed). The five mismatches in it, comparing to the typical p53-binding site, were capitalised and underlined. Another probe representing the sequence in the albumin gene served as the negative control, which was amplified from genome DNA by PCR using the forward and reverse primers, 5′-GCTGTCATCTCTTGTGGGCTGT-3′ and 5′-ACTCATGGGAGCTGCTGGTTC-3′. The probes were generated by annealing the forward and reverse oligonucleotides, followed by end labelling using T4 polynucleotide kinase in the presence of [*γ*-^32^P]dATP. To perform EMSA, extracts were prepared from MCF7/AdrR and MCF7/WT cells as described (Tian *et al*, 2005a, 2005b). A total of 10 *μ*g nuclear extract and 50 ng of p53 protein were applied to perform EMSAs. The detailed procedure for carrying out EMSAs was described previously (Tian *et al*, 2005b). Super-EMSA experiments were performed in the presence or absence of 1 *μ*g of monoclonal p53 antibody (p53-(DO)-1, Santa Cruz) for 30 min at room temperature and analysed on a 4% non-denaturing-PAGE gel. The experiments were repeated three times.

### ChIP assays

MCF7/WT cells were treated with 1 *μ*M DOX for 20 h to induce *p53* expression. Chromatin immunoprecipitation assays were carried out as described previously (Adachi *et al*, 2004). Briefly, genomic DNA and proteins were crosslinked by the addition of 1% final concentration of formaldehyde directly into the culture medium and incubated for 30 min at 37°C. Cells were lysed and sonicated to 300–1000 bp DNA fragments. After centrifugation, the supernatant was diluted 10 times with the ChIP buffer and incubated with the agarose conjugated with anti-p53 (p53-AC) or -HA (negative control) antibodies (Santa Cruz) at 4°C overnight. Immune complexes were precipitated and washed. The DNA–protein complexes were decrosslinked by heating at 65°C for 5 h with high concentration salt and DNA was purified and resuspended in 50 *μ*l of TE buffer. The input DNA was diluted 100 times before PCR. The bound and input DNAs were analysed by PCR (35 cycles). Specific primers for the *p53* response element in the *WTH3* promoter were 5′-GCCCTAGCCTTGGGAAGCCAAAG-3′ (forward) and 5′-CGGCAGAGTAGCCGAGCACG-3′ (reverse). The sense and anti-sense primers for *p21* (positive control) and albumin (negative control) promoters were 5′-GTGGCTCTGATTGGCTTTCTG-3′ and 5′-CTGAAAACAGGCAGCCCAAG-3′ as well as 5′-GCTGTCATCTCTTGTGGGCTGT-3′ and 5′-ACTCATGGGAGCTGCTGGTTC-3′.

### 4′,6-Diamidino-2-phenylindole (DAPI) staining assays

Hela cells were seeded onto glass cover slips placed in 6-well plates. When the cells reached 50% confluence, they were transfected with pcDNA3.1/WTH3 or pcDNA3.1 in parallel using Lipofectamine™ 2000 (Invitrogen, Carlsbad, CA, USA) as described previously (Shan *et al*, 2000, 2002a) following the manufacturer's instructions. Nuclear staining with DAPI was performed as described (Kim *et al*, 2002). After 24 h of transfection, cells were washed with 1 × phosphate-buffered saline (PBS), fixed with 70% ethanol and washed again with PBS. The cells then were treated with DAPI (1 *μ*g/ml) (Sigma) for 12 min, washed with PBS for 5 min, and treated with VectaShield (Vector Laboratories, Burlingame, CA, USA). Stained nuclei were visualised under a fluorescent microscope. Apoptotic cells were morphologically defined by cytoplasmic and nuclear shrinkage and chromatin condensation. The experiments were repeated three times.

### Terminal deoxynucleotidyl transferase biotin-dUTP nick end labelling (TUNEL) assays

Hela and HEK293 cells were seeded in 6-well plates. When the cells reached 50% confluence, they were transfected with pcDNA3.1/WTH3 or pcDNA3.1. After 18 (Hela) and 30 (HEK293) hours of transfection, cells were trypsinised and washed with 2 × PBS and transferred onto a glass slide and air dried. The cells were fixed in 3% paraformaldehyde for 30 min and washed in PBS. Terminal deoxynucleotidyl transferase biotin-dUTP nick end labelling assays were performed using the FragEL™ DNA Fragmentation Detection Kit (Calbiochem, San Diego, CA, USA) following the manufacturer's protocol. The apoptotic cells, which exhibited a brown stain, were visualised under a microscope. The experiments were repeated three times.

### Flow cytometry

Approximately 5 × 10^5^ cells/well of HEK293 were transfected with zero or equal mole ratio of pcDNA/WTH3 or pcDNA3.1. The pCMV/*β*-galactosidase was also transfected in parallel as a transfection efficiency control. After 24 h of transfection, the cells were harvested by trypsinisation, washed in PBS, and the DNA was stained with propidium iodide (PI, 50 *μ*g/ml) containing 250 *μ*g/ml of ribonuclease A, followed by flow cytometry analysis as described previously (Shan *et al*, 2002a). The experiments were repeated three times.

## RESULTS

### The *WTH3* promoter was positively regulated by *p53*

**Figure 1:**
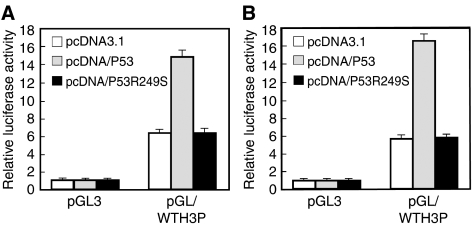
Results obtained from luciferase assays where pGL3 or pGL/WTH3P was cotransfected with pcDNA3.1, pcDNA/P53 or pcDNA/P53R249S into (**A**) MCF7/WT and (**B**) HEK293 cells. Relative luciferase activities driven by the *WTH3* promoter under the influence of the empty vector, wild-type and mutated *p53* were compared.

Since results generated by the Patch Search indicated that the p53M sequence in the *WTH3* promoter could be a potential binding site for p53, we speculated that the *p53* gene could regulate the *WTH3* promoter. To test this hypothesis, pGL/WTH3P or the empty vector, pGL3, was cotransfected with pcDNA3.1, pcDNA/P53 or pcDNA/P53R249S, as well as pCMV/*β*-galactosidase into MCF7/WT and HEK293 cells. The enzyme activities driven by the *WTH3* promoter under the influence of wild-type and mutated *p53* were measured with justification of protein concentrations and transcription efficiency (Figure 1A and B). We found that the wild-type *p53*, but not its mutant, increased *WTH3* promoter activity approximately 2.5–3 times in both hosts. As the *TSP50* gene, which was negatively regulated by *p53*, was available in our lab, we cotransfected pGL/TSP50P (pGL containing the *TSP50* promoter) with pcDNA/P53, in parallel, into MCF7/WT and HEK293 cells. The results clearly showed that *p53* downregulated the *TSP50* promoter (Xu *et al*, 2007), whereas it upregulated the *WTH3* promoter. Similar results were obtained when MCF7/AdrR and Hela cells were used as hosts (data not shown), which suggested that *WTH3* was upregulated by the *p53* gene in a cell-type independent manner. This information provided us with a plausible explanation that *WTH3*'s low expression observed in MCF7/AdrR could be the result of *p53* dysfunction owing to the mini deletion in its DNA-binding domain, whereas MCF7/WT, which contains the wild-type *p53* gene, expressed a relatively high level of *WTH3* (Norbury and Zhivotovsky, 2004). To examine further the correlations between these two genes, we measured *WTH3* gene expression levels in a cell line panel with defined *p53* gene status.

### There was a correlation between *WTH3* expression levels and *p53* gene status

**Figure 2:**
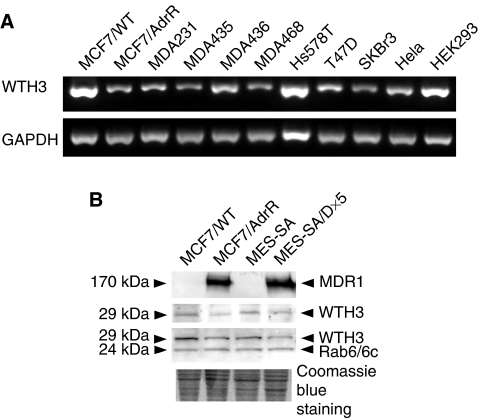
(**A**) Results obtained from SQRT-PCR to estimate endogenous *WTH3* gene expressions in 11 cell lines with defined *p53*-gene status. (**B**) Western blot analysis of WTH3 proteins in MCF7/AdrR *vs* MCF7/WT and MES-SA/D × 5 *vs* MES-SA using WTH3 (1 : 200 dilution) and Rab6 (1 : 2000 dilution) antibodies. In addition, MDR1 detected by the MDR1 antibody (1 : 30 dilution) served as one of the controls. Proteins and their molecular weights are indicated on the right and left. Identical SDS-PAGE gels with the same amount of protein (100 *μ*g/lane) were loaded. Three gels were used for Western blot by three antibodies; the fourth gel was stained with Coomassie blue for protein concentration normalisation.

*WTH3* expression levels in nine breast carcinoma cell lines, Hs578T, MCF7/AdrR, MCF7/WT, MDA-MB-231, MDA-MB-435, MDA-MB-436, MDA-MB-468, T47D and SKBr3, as well as in Hela and HEK293 were examined by SQRT-PCR. The results showed that MCF7/WT and HEK293, which contained the wild-type *p53* gene, produced the highest *WTH3* RNA as compared to the remaining cell lines whose *p53* gene was either mutated, as in Hs578 T, MCF7/AdrR, MDA-MB-231, −435, −436, −468, T47D and SKBr3, or attenuated, as in Hela cells by the papillomavirus E6 protein that targets p53 for degradation (Li *et al*, 2004) (Figure 2A). Although each cell line contains its own unique, complicated biological characteristics, the results showed a trend that the cell lines possessing mutated *p53* expressed relatively low *WTH3* RNA as compared to those containing its wild type. However, we will further confirm this correlation via other approaches in the future. In addition, reduced *WTH3* transcripts were also reflected at the protein level when antibodies for WTH3 and Rab6 were used to measure the WTH3 protein in MCF7/AdrR *vs* MCF/WT and MES-SA/D × 5 *vs* MES-SA cells. Densitometer analysis showed that the protein amount detected by the WTH3 antibody was significantly higher in MCF7/WT and MES-SA as compared to their MDR counterparts (Figure 2B). Rab6 antibody, which recognised both Rab6c (a housekeeping gene, served as the quantitative control) and WTH3 protein, generated similar results. This was consistent with previous data that found *WTH3* transcript levels in MCF7/WT and MES-SA were much higher than those in MCF7/AdrR and MES-SA/Dx5 (Shan *et al*, 2002a). Since both MDR cell lines expressed extremely high levels of the *MDR1* gene relative to their corresponding parental cell line, the MDR1 antibody was also used to detect MDR1 protein levels in those cells, which served as another control.

### The *p53* transgene elevated endogenous *WTH3* gene expression

**Figure 3:**
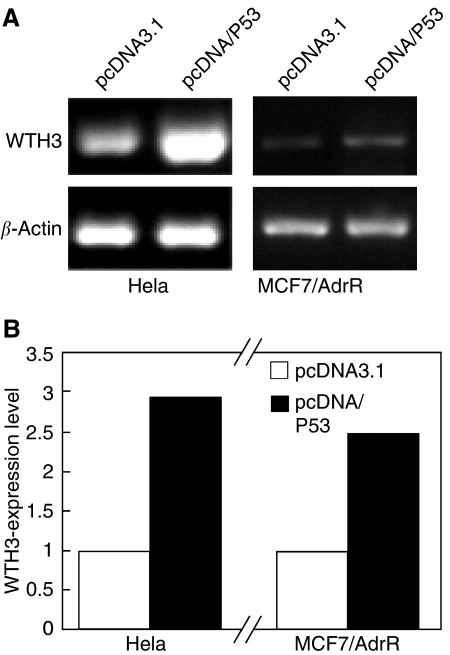
Results obtained from SQRT-PCR to estimate endogenous *WTH3* gene expressions in Hela and MCF7/AdrR cells, which were influenced by the *p53* transgene. (**A**) The two cell lines were either transfected with pcDNA3.1 or pcDNA/P53, where *β*-actin served as the quantitative control. (**B**) Quantitative comparison of the results presented in (**A**). The empty and black columns represent *WTH3* expression levels under the influence of the empty vector or the *p53* transgene, respectively.

Both Hela and MCF7/AdrR cells were used as the hosts for *p53* gene transfection. Briefly, pcDNA/P53 or pcDNA3.1 and pCMV/*β*-galactosidase were introduced into the cells. After 24 h, total RNA was isolated from the transfectants and SQRT-PCR was performed to evaluate the amount of *WTH3* transcripts. As expected, cells expressing the *p53* transgene generated approximately 2.5 times higher *WTH3* transcripts than the control cells (Figure 3A and B). Taken together, the data gathered from performing different experiments suggested that *WTH3* could be a target of the *p53* gene. To gain detailed information on how *p53* controlled *WTH3* gene expression, we next wanted to identify the p53-response element in the gene promoter by creating serial deletion mutants.

### The region (−396 to −289) in the *WTH3* promoter contained the p53-response element

**Figure 4:**
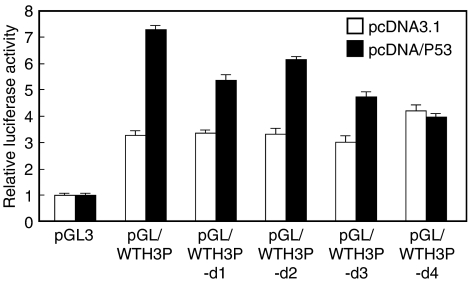
Luciferase assay results for the *WTH3* promoter deletion mutants under the influence of the *p53* gene in HEK293 cells. The empty and black columns represent relative luciferase activity driven by the empty vector (pGL3), wild-type *WTH3* and four deleted promoter mutants (pGL/WTH3P-d1, -d2, -d3 and -d4), which were either influenced by the empty vector (pcDNA3.1) or the *p53* transgene.

Since p53M contained five mismatches, we were not sure if it was the real p53-binding site. Instead of generating a targeted deletion mutant, we created six serial deletions (each was ∼100 bp shorter than the adjacent one) by PCR amplification using pGL/WTH3P as the template. The resulting PCR products were constructed into pGL3 to obtain pGL/WTH3P-d1 to -d6 constructs. Each of the plasmids was then cotransfected with pcDNA/P53 or pcDNA/3.1 along with pCMV/*β*-galactosidase. After 24 h, the luciferase activity driven by the wild-type and mutated promoters under the influence of *p53* was determined. We found that pGL/WTH3P-d4 no longer responded to the *p53* transgene as its enzymatic activity was similar to that in the control cells that were transfected with pGL3 and pcDNA/P53. This finding suggested that the deleted region (from −396 to −289) could contain the p53-response site (Figure 4). As the p53M sequence resided in this region, we believed it could be a direct target of the p53 protein. To test this possibility, EMSA and super-EMSA assays were performed.

### The p53 protein bound to the *WTH3* promoter in the region from −330 to −282

**Figure 5:**
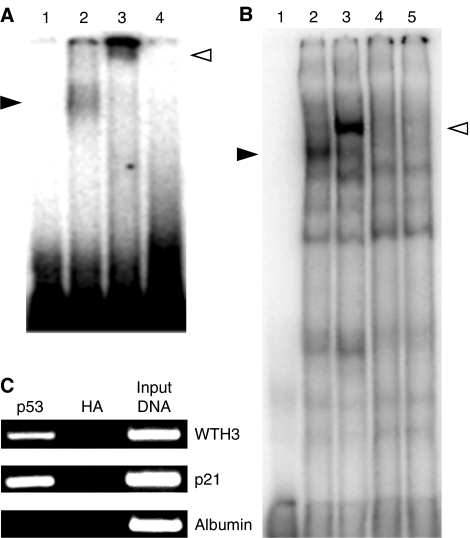
(**A**) Results obtained from EMSA and super-EMSA assays where P49 was used as the probe that was interacted with the purified p53 protein. Lane 1 contains the labelled probe. Lane 2 contains the labelled probe and 50 ng of p53 protein. The solid arrow indicates the DNA/p53 complexes. Lane 3 contains the labelled probe, 50 ng of p53 protein and the anti-p53 antibody. The empty arrow indicates the antibody shifted DNA/p53 complexes. Lane 4 contains the labelled probe, 50 ng of p53 protein and 100 times excess of cold P49. (**B**) Results obtained from EMSA and super-EMSA assays where the P49 probe and nuclear extracts obtained from MCF7/AdrR and MCF7/WT were used. Lane 1 contains the labelled probe. Lane 2 contains the labelled probe and 10 *μ*g of nuclear extract prepared from MCF7/WT cells. The solid arrow indicates the DNA/p53 complexes. Lane 3 contains the labelled probe, 10 *μ*g of MCF7/WT nuclear extract and the anti-p53 antibody. The empty arrow indicates the antibody shifted DNA/p53 complexes. Lane 4 contains the labelled probe, 10 *μ*g of nuclear extract prepared from MCF7/AdrR cells. Lane 5 contains the labelled probe, 10 *μ*g of MCF7/AdrR nuclear extract and the anti-p53 antibody. (**C**) Chromatin immunoprecipitation assay using MCF7/WT cells treated with 1 *μ*M DOX. p53–DNA complexes were captured with anti-p53 antibody. Anti-HA antibody was used as the negative control. PCR products representing the *WTH3, p21* (positive control) and *albumin* promoter (negative control) sequences are noted.

A 49 bp probe (from −330 to −282), P49, which contained the p53M sequence, and an albumin genomic DNA sequence (negative control) were utilised to perform EMSA assays. After isotope labelling, the probes were first incubated with purified p53 protein. The DNA/protein complex was realised on a nondenaturing-PAGE gel. We found that the P49 probe, but not the control probe (data not shown), interacted with the p53 protein. This interaction was specific as the p53 antibody shifted the P49/protein complex into a much higher position and excessive amounts of the cold P49 probe competed away p53 from the complex (Figure 5A). In addition, the same strategy was applied where P49 and nuclear extracts prepared from paired MCF7 cells were used. The results showed that the P49 probe interacted with the p53 proteins generated from MCF7/WT, but not the mutated ones in MCF7/AdrR cells (Figure 5B). However, no forms of the p53 proteins bound to the negative control probe (data not shown) (Figure 5B). Proteins of both sources did not bind to the negative control probe (data not shown). To confirm further that the p53 protein was indeed involved, supershift EMSA was performed utilising p53 antibody. We found that the p53 antibody only shifted the P49-MCF7/WT-protein complex into a much higher position (Figure 5B). To understand further if p53M was the direct target of the p53 protein, three probes were generated, which represented 28 bp of the right (P49R, containing p53M), 36 bp of the left (P49L) and 31 bp of the centre (P49C, from 10 to 40) of the P49 sequence, respectively. The EMSA results showed that the p53 protein did not bind to any of them (data not shown), which suggested that the whole P49 sequence was essential for interacting with p53. To verify further that *WTH3* was a direct target of the *p53* gene, ChIP assays were carried out.

### The p53 protein bound to the *WTH3* promoter *in vivo*

Usually, under normal condition, *p53* gene expression is relatively low in cells. However, when responding to DNA-damaging reagents, *p53* transcripts are significantly increased. The elevated gene product either functions as a transcription factor for its targeted genes or directly performs its cellular roles, such as promoting apoptosis. Here, we tested whether *p53* could directly target the *WTH3* promoter in MCF7/WT cells. To this end, the cells were treated with DOX over different time courses to induce endogenous *p53* gene expression. Western blot analysis determined that during 8–24 h treatment period, similar amounts of *p53* expression, elevating to approximately 10 times the original level, were exhibited (data not shown). Next, the p53–DNA complexes were immunoprecipitated with anti-p53 or anti-HA antibodies. To see if the *WTH3* promoter containing the p53-response site was in the enriched DNA fragments, PCR was performed. The upstream sequences of the *p21* and *albumin* promoters, which were with or without a p53-response element, were also amplified and served as the positive and negative control, respectively. The results showed that both *WTH3* and *p21*, but not *albumin*, promoter regions were enriched by the p53 antibodies. In addition, none of the promoter regions were enriched by the HA antibodies (Figure 5C). These findings suggested that *WTH3* was a direct target of the *p53* gene. As past research demonstrated that confirmed *p53* target genes either are *p53* functional mediators, such as *p21, Bax* and *PUMA*, or *p53* functional regulators, such as *MDM2, COP1* and *PML* (Liu and Chen, 2006), we then examined if *WTH3* and *p53* shared some biological functions. To date, several approaches were employed to test if *WTH3* played a role in promoting apoptosis.

### Overexpression of *WTH3* induced apoptotic nuclear condensation

**Figure 6:**
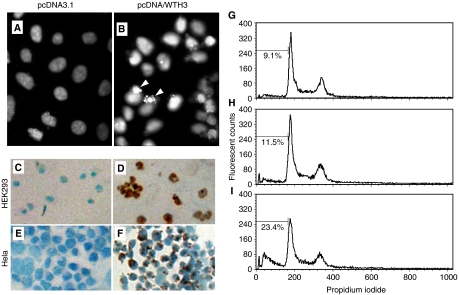
(**A** and **B**) results obtained from DAPI staining assays using Hela cells. In (**A**) and (**B**) Hela cells contain pcDNA3.1 and pcDNA/WTH3, respectively. Cells were stained with DAPI, and the stained nuclei were visualised under a fluorescent microscope (magnification, × 400). Arrows indicate apoptotic nuclear condensation. (**C**–**F**) results obtained from TUNEL assays using Hela and HEK293 cells as hosts. (**C** and **D**) HEK293 cells were transfected with pcDNA3.1 and pcDNA/WTH3, respectively, for 30 h. (**E** and **F**) Hela cells were transfected with pcDNA3.1 and pcDNA/WTH3, respectively, for 18 h. DNA fragmentation in the apoptotic cells induced by the *WTH3* gene were stained with a brown colour as compare to the control cells containing the empty vector, which were blue in colour. (**G**–**I**) results generated by flow cytometry assays using HEK293 cells. (**G**) Cells served as vehicle control. (**H** and **I**) cells were transfected with pcDNA/3.1 and pcDNA/WTH3, respectively. The cells were then stained with PI and the intercellular fluorescence was measured by a flow cytometer. The sub-G1 DNA content in each group, which represented apoptotic cells, is indicated by a percentage.

To test whether *WTH3* could cause cell death, pcDNA/WTH3 was transiently introduced into Hela cells. After 24 h, the transfectants containing the exogenous *WTH3* gene or empty vector were treated with DAPI, a fluorescent DNA-binding dye. The cells' nuclear morphology was examined under a fluorescent microscope. The typical morphological features of apoptotic cells were observed in the population of cells transfected with pcDNA/WTH3, but not in the control cells transfected with the empty vector (Figure 6A and B). These findings indicated that the *WTH3* gene-induced apoptosis in Hela cells.

### Overexpression of *WTH3*-induced cell death

To confirm further *WTH3*'s apoptotic potential, TUNEL assays were carried out using both HEK293 and Hela cells as hosts. After receiving pcDNA/WTH3 or pcDNA3.1, TUNEL staining was performed. We found that both host cells transfected with the *WTH3* transgene displayed brown colour staining that indicated a typical apoptotic condition, whereas the corresponding controls were stained with blue colour (Figure 6C–F). These findings were consistent with the results of the DAPI staining assays. Thus, *WTH3*-induced apoptosis in the two cell lines tested.

### Overexpression of *WTH3* increased sub-G1 cell population

HEK293 cells, which exhibited the highest transfection efficiency, were transfected with pcDNA/WTH3 or pcDNA3.1 to perform flow cytometry assays. By measuring the cells with sub-G1 DNA content, which is believed to represent apoptotic cells, we found that after 24 h, 23.4% of the cell population had under gone apoptosis after receiving the pcDNA/WTH3 construct. However, only 11.5 and 9.1% of the cell population underwent apoptosis after receiving the pcDNA3.1 vector and vehicle control, respectively (*P*<0.01) (Figure 6G–I). These results further suggested that the *WTH3* gene stimulated apoptosis.

## DISCUSSION

The *WTH3* gene was discovered owing to its hypermethylation in MCF7/AdrR cells. Earlier studies suggested that it was a negative regulator for MDR development (Shan *et al*, 2002a; Tian *et al*, 2005a, 2005b), which made it extremely interesting as most MDR-related genes discovered so far exert a positive effect. To understand the mechanisms involved in its downregulation in MDR cells the gene's promoter was identified and analysed (Tian *et al*, 2005b). We found that it was differentially regulated in MCF7/AdrR and MCF7/WT cells. Several mechanisms could be involved in this differential regulation, which included drug-induced epigenetic modifications and alteration of *trans*-elements, we believe this is the situation as the *WTH3* promoter was found to be hypermethylated in cultured and primary drug resistant cells, and a region targeted by DNA methylation and a repeat sequence in the promoter interacted with diverse transcription complexes prepared from MCF7/AdrR *vs* MCF7/WT (Tian *et al*, 2005a, 2005b).

To gain more detailed information regarding the *WTH3* gene's differential regulation, we performed the Patch Search, which led to the discovery of a p53-binding motif, p53M. The *p53* gene is an important tumour suppressor and is involved in apoptosis and cell cycle arrest, whereas mutations of *p53* is associated with human cancers and the onset of MDR in a broad range of malignancies (Schmitt and Lowe, 1999; Norbury and Zhivotovsky, 2004; Pommier *et al*, 2004; Kim, 2005). Discovery of a putative p53-binding site in the *WTH3* promoter led us to explore whether *p53*-regulated *WTH3* expression. By performing luciferase assays we found that the *p53* transgene significantly elevated promoter activity in all the host cell lines tested. In addition, evaluating *WTH3*'s expression levels in 11 cancer cell lines with defined *p53* status supported this positive influence as the cell lines with wild-type *p53* produced much higher levels of *WTH3* transcript than those containing mutated or attenuated *p53*. We also noticed that the observed correlation between these two genes in breast cancer cell lines was not related to their oestrogen receptor positive or negative condition. However, whether *WTH3* gene activity coincides with the degree of malignancy remains to be determined. Further observations revealed that the *p53* transgene was able to considerably increase endogenous *WTH3* gene expression in both Hela and MCF7/AdrR cells, which supported the possibility that *WTH3* could be a target of *p53.* This hypothesis turned out to be true as EMSA and super EMSA assays demonstrated that the P49 probe containing p53M in the *WTH3* promoter was specifically targeted by endogenous and purified p53. However, p53 with mini deletion in MCF7/AdrR cells, which lost its DNA-binding capability, did not bind to the probe. In addition, ChIP assays confirmed that the p53 protein physically interacted with the *WTH3* promoter region that contained the P49 sequence. In addition, it is worth to mention that EMSA results showed that the p53 protein was not able to bind to the probe that only included the GC rich region, the p53M site, or the sequence containing part of the GC and p53M regions, which suggested that both GC rich and p53M sequences were required for *p53* gene targeting. We also noticed that the GC rich region included three CpG sites that were differentially methylated in MDR *vs* non-MDR cells (Tian *et al*, 2005a, 2005b). The DNA methylation is one of the epigenetic modifications that is symbolised by reversing traits of gene expression without DNA sequence change (Doerfler, 1983; Bird, 1986; Antequera and Bird, 1993a, 1993b; Kass *et al*, 1997; Siegfried and Cedar, 1997). At present, little is known about how the epigenetic network interacts with other transcriptional machineries to regulate gene expression in mammalian cells. In the past, p53-binding motifs with GC rich features were observed in several gene promoters including *EGFR, Killer/DR4, RB* and *TGF-α* (Qian *et al*, 2002). However, there are fundamental questions that need to be answered. For example, are those GC-rich regions epigenetically modified? If they are, do they exert a negative impact on p53-transactivity? As the p53-response element in the *WTH3* gene promoter was involved in differential methylation, we have been provided with a unique working model system that can possibly answer those questions. Currently, we are designing experiments to explore if there is any interplay between DNA methylation and the p53 transcription factor in regulating *WTH3* gene expression.

Prior research demonstrated that confirmed *p53* target genes are either *p53* functional mediators or regulators (Liu and Chen, 2006). Considering that *WTH3* is a target of the *p53* gene, we examined if they shared some biological functions. By employing several strategies, we found that *WTH3* played a role in promoting apoptosis. It is possible that this proapoptotic potential is the driving force behind *WTH3*'s participation in MDR development. In addition, as WTH3 is a G protein and most likely involved in cellular signalling transduction, we cannot rule out another prospect that it also acts as a *p53* functional regulator. Testing this hypothesis is one of the subjects of our future research.
